# A community health promotion project: Amazing Race for Heart Health

**DOI:** 10.3389/fepid.2023.1278672

**Published:** 2023-12-20

**Authors:** Jessica A. Reese, Carla Guy, Halana Jay, Tauqeer Ali, Elisa T. Lee, Ying Zhang

**Affiliations:** ^1^ Center for American Indian Health Research, Hudson College of Public Health, University of Oklahoma Health Sciences Center, Oklahoma City, OK, United States; ^2^ Department of Biostatistics and Epidemiology, Hudson College of Public Health, University of Oklahoma Health Sciences Center, Oklahoma City, OK, United States

**Keywords:** cardiovascular disease, cardiovascular education, Strong Heart Study, American Indian, cardiovascular disease prevention, community health promotion

## Abstract

**Introduction:**

American Indians have higher rates of cardiovascular disease (CVD), likely due to disproportionate burden of diabetes and limited access to widespread CVD prevention programs such as Honoring the Gift of Heart Health (HGHH), a 10-week CVD risk factor awareness curriculum. Due to its length, HGHH may be difficult to complete; therefore, we aimed to evaluate a shortened CVD risk factor awareness program based on the HGHH educational materials for American Indians residing in southwest Oklahoma, entitled “The Amazing Race for Heart Health.”

**Methods:**

We conducted an interventional study where each participant served as their own control (*n* = 61), with pre- and post-intervention measurements. We included American Indians from seven tribal nations in southwest Oklahoma. At two interventional meetings we used educational materials and activities from HGHH focusing on nutrition, cholesterol, diabetes, hypertension, physical activity, and heart attack warning signs. McNemar's test was used to determine the effectiveness of the intervention on raising CVD risk factor awareness.

**Results:**

When comparing the pre- and post-survey responses, the percentage of correct responses either stayed the same or increased. Knowledge improved in 11/25 (44%, *p* < 0.05) domains including the difference between good and bad cholesterol and types of physical activity that can prevent CVD. When considering diabetes, knowledge increased regarding the interaction between diabetes and cholesterol in the association with CVD.

**Discussion:**

These results demonstrate that the “Amazing Race for Heart Health,” a shortened CVD risk factor educational program based on the HGHH educational materials, was effective at increasing awareness regarding CVD risk factors.

## Introduction

Cardiovascular disease (CVD) among American Indians was once thought to be low; however, results from the Strong Heart Study (SHS) have demonstrated that CVD in American Indians is higher than other United States (US) populations (1–3) and more likely to be fatal (1–4). Higher CVD prevalence and mortality in American Indian communities are likely due to disproportionate burden of diabetes, (5, 6) socioeconomic barriers to comprehensive cardiovascular care, (7) and limited access to widespread CVD prevention programs (8). Of the few programs that have been implemented in American Indian communities, many have focused health education on reducing body weight to achieve a proposed target to reduce the risk of diabetes and CVD (9, 10). In addition, most of these strategies have focused on American Indians with higher risks (i.e., metabolic syndrome), (9, 10) rather than the general American Indian populations residing in communities. Since culturally tailored CVD risk factor education is an integral component to intervention techniques for disease prevention, (11, 12) it is imperative that educational along with lifestyle interventions be expanded beyond weight control and be applicable on the community level (9, 10). Therefore, the proposed target for this study included education of additional CVD risk factors (beyond weight control), and implementation among a study population that was selected to be similar to the general population of American Indians residing in Southwest Oklahoma (i.e., not restricted to high risk groups). These proposed targets were based on focus group discussions with community members prior to developing the intervention.

To confront the high rates of CVD, (2, 13) potential interventions should be developed with direct community involvement (11, 12). Researchers, tribal nations, and community members must work collaboratively in decision-making process to develop a culturally relevant lifestyle intervention to reduce CVD risk (11, 14, 15). “Honoring the Gift of Heart Health” (HGHH) is a CVD risk factor education program developed by the National Heart, Lung, and Blood Institute for use in American Indian communities (16, 17). HGHH is unique because it incorporates a family-oriented approach for adopting heart health recommendations by emphasizing lifestyle changes for people of all ages (16). Although previous research indicates that HGHH has been effective, (18, 19) the full 10-week program may be difficult for participants to complete, due to working schedule conflicts, caring for family members, and other life priorities. Therefore, we aimed to work with American Indians residing in southwest Oklahoma to implement and evaluate a shortened CVD risk factor educational intervention program based on the HGHH materials.

## Methods

### Educational intervention development

To develop the shortened educational intervention based on HGHH materials, first we worked with the American Indian communities using a qualitative research design and a narrative inquiry approach, which has been previously described (20). Briefly, we conducted three focus group discussions, with 12 total participants. Two researchers transcribed the focus group audio recordings and conducted a content analysis to identify themes and patterns. American Indian participants provided recommendations regarding improving cultural responsiveness of the HGHH. On average, participants spent the highest percentage of time discussing themes related to nutrition (46%), followed by educational material format, risk factors, special groups, and new recommendations (20). This part of the project was the first step in working with American Indian communities to design a more effective and relevant educational intervention (11, 20). Next, we worked with a tribal liaison and other members of the community to incorporate the recommendations and to conduct the intervention in the communities.

### Study population and recruiting

To determine if the intervention strategy was effective, we conducted a study where each participant served as their own control, with pre- and post-intervention survey measurements. We included American Indians from seven tribal communities that participated in the Strong Heart Study (SHS), which is a prospective cohort study of CVD in American Indians. Members of these tribal communities primarily reside in the rural areas of southwest Oklahoma; however, some reside in the urban areas of Oklahoma City. Throughout 2022, a tribal liaison (CG) and SHS field staff (HJ) used a snowball sampling approach to recruit participants for two separate interventional meetings. Tribal liaisons are important because they are tribal members that have leadership roles within the community. Therefore, they effectively communicate with the tribes and their members while respecting tribal sovereignty. Since the tribal liaison and the SHS field staff are members of the tribal communities, initial recruitment was based on their existing relationships within the community.

The first meeting included members of communities residing in Oklahoma City and was conducted at the University of Oklahoma Health Science Center. The second meeting included American Indian residents from SHS communities in rural areas and was conducted at the Wichita and Affiliated Tribes Community Center in Anadarko, Oklahoma. This study was approved by the participating tribes, University of Oklahoma Institutional Review Board, and the Oklahoma City Area Indian Health Service Institutional Review Board. We obtained written informed consent from each participant and community approval from the joint Southwest Oklahoma Intertribal Health Board.

### Assessments

After obtaining individual informed consent, we asked the participants to complete a brief demographic survey, a heart disease risk factor assessment, and a heart disease risk factor knowledge survey (21). With the demographic survey, we collected age, gender, tribal affiliation, area of residence (urban or rural), and the highest attained level of education. The heart disease risk factor assessment contained self-reported CVD risk factors including current smoking behaviors, high blood pressure, high cholesterol, diabetes, being overweight or obese, being physically inactive, and having a family history of heart disease (17). Finally, we administered the heart disease risk factor knowledge survey. This survey was previously developed and validated and is a 25-item questionnaire that measures risk factor knowledge in people with diabetes (21). Although this questionnaire was not developed specifically for American Indians, it is relevant because of the high prevalence of diabetes in the American Indian communities that participate in the Strong Heart Study (22, 23). To determine if the intervention changed the participants' understanding of CVD risk factors, we administered the risk factor knowledge survey before and after the educational intervention.

### Amazing race or heart health educational intervention

We delivered the intervention at two separate meetings. We began meetings with informal introductions, then we obtained individual informed consent and administered the assessments including the risk factor knowledge pre-survey. Next we delivered a 20 min educational power point presentation based on materials from the HGHH curriculum (16). We focused on HGHH topics that were interesting and relevant, based on comments during the focus groups that were previously conducted (17, 20). We presented information on the difference between saturated, unsaturated, and trans fats, describing what types of foods contain which types of fats. We then discussed how fat is related to blood cholesterol and the difference between low-density and high-density lipoproteins. Following the discussion on cholesterol, we discussed information on hypertension and hypertension medications. Then, we discussed diabetes, the amount of sugar in different types of food, and the relationship between sugar consumption and diabetes control. We defined diabetes, its symptoms, how it is related to heart disease, and the risk factors associated with diabetes. Then, we discussed some simple activities that participants can do to increase their physical activity, such as walking and gardening. Finally, we reviewed the heart attack warning signs presented in HGHH (16, 17).

To reinforce the information from the interactive presentation, we created the “Amazing Race for Heart Heath,” using activities and handouts from HGHH (17). During this part of the intervention, 6 groups of 5–6 people participated in an “scavenger hunt,” which lasted approximately 45 min. The participants performed the HGHH activities listed below:

1. *Nutrition:* We asked participants to choose the lower calorie food item from “Sally's Snack Choices.” In addition, we asked the participants to calculate the number of calories that Sally would save if she chose the lower calorie option.
2. *Cholesterol:* We asked participants to guess the amount of fat in various food items.
3. *Diabetes:* We asked participants to guess the amount of sugar in a big gulp, a bag of skittles, and a snickers bar. We had props including the actual candies and Styrofoam cup that would hold a big gulp. In addition, we had the amount of sugar contained in these items in a plastic bag.
4. *Hypertension:* We asked participants to list the four types of hypertension medications and how they work.
5. *Physical activity:* For this station, we asked the participants to complete the stretching exercises located in HGHH educator's manual. Then we asked them to walk a short distance.
6. *Heart attack warning signs:* Finally, we asked the participants to name the six heart attack warning signs located in HGHH educator's manual.

After successfully performing each of these activities, participants were given hints about where to go next. They also received handouts and materials to take home, based on HGHH educational materials. At the end of the race, we awarded study t-shirts to the group finishing first. Finally, we administered a post-survey to determine if they learned and retained the material (21).

### Data analysis

We used descriptive statistics to summarize the population, calculating median and range for age and percents for all other variables. To assess CVD risk factor knowledge, we calculated the percent who correctly responded to each question on the pre- and post-surveys and used McNemar's test to determine if the responses on the surveys differed (24). We used SAS Software, version 9.4 for analyses (25).

## Results

### Study population demographics

We recruited 61 American Indian participants with a median age of 55 years (range = 23–88); 79% were female, 48% had ≥12 years of education, and 51% lived in rural Oklahoma. Regarding CVD risk factors, about one-half of the study population reported being overweight/obese (53%) or having hypertension (46%). Roughly one-third of the participants reported having high cholesterol (26%), diabetes (30%), physical inactivity (30%), or a family history of CVD (38%). Finally, 16% reported current smoking (Table 1).

**Table 1:** Demographics and cardiovascular disease risk factors of American Indian study participants from southwestern Oklahoma (*n* = 61).

Variable	Median (range) or percentage
Age (years)	55 (23–88)
Gender (% female)	78.7
Area of residence (% rural)	51
Completed Education	
High School	36.1
More than High School	47.5
Prefer not to answer	16.4
Current Smoking (% yes)	16.4
High Blood Pressure (% yes)	45.9
High Cholesterol (% yes)	26.2
Diabetes (% yes)	29.5
Overweight or obese (% yes)	52.5
Physically inactive (% yes)	29.5
Family History of Heart Disease (% yes)	37.7

### CVD risk factor knowledge

Since each participant served as their own control, we collected 122 observations from the pre- and post- CVD risk factor knowledge surveys. When comparing the pre- and post-surveys, the percentage of correct responses either increased or stayed the same. The participants had good baseline knowledge of several risk factors being associated with heart disease, including age (question 3), smoking (questions 4 and 5), high blood pressure (questions 6 and 7), being overweight (question 12), and having diabetes (questions 16–18, Table 2).

**Table 2:** Heart disease risk factor knowledge survey responses before and after an educational intervention for American Indians from communities that participate in the Strong Heart Study in southwest Oklahoma.

Question	Correct	Correct on Pre-survey (%)	Correct on Post-survey (%)	*p*-value
1. A person always knows when they have heart disease	False	78.9	84.6	0.32
**2. If you have a family history of heart disease, you are at risk for developing heart disease**	**True**	**73**.**1**	**88**.**5**	**0**.**03**
3. The older a person is, the greater their risk of having heart disease	True	50.0	61.5	0.08
4. Smoking is a risk factor for heart disease	True	92.3	96.2	0.31
5. A person who stops smoking will lower their risk of developing heart disease	True	78.9	86.5	0.25
6. High blood pressure is a risk factor for heart disease	True	96.2	94.2	0.56
7. Keeping blood pressure under control will reduce a person's risk for developing heart disease	True	84.6	92.3	0.16
**8. High cholesterol is a risk factor for developing heart disease**	**True**	**75**.**0**	**90**.**4**	**0**.**02**
9. Eating fatty foods does not affect blood cholesterol levels	False	86.5	75.0	0.11
**10. If your “good” cholesterol (HDL) is high you are at risk for heart disease**	**False**	**28**.**9**	**61**.**5**	**<0**.**01**
**11. If your ‘bad’ cholesterol (LDL) is high you are at risk for heart disease**	**True**	**65**.**4**	**80**.**8**	**0**.**03**
12. Being overweight increases a person's risk for heart disease	True	90.4	92.3	0.65
**13. Regular physical activity will lower a person's chance of getting heart disease**	**True**	**75**.**0**	**90**.**4**	**0**.**02**
**14. Only exercising at a gym or in an exercise class will lower a person's chance of developing heart disease**	**False**	**73**.**1**	**86**.**5**	**0**.**03**
**15. Walking and gardening are considered exercise that will help lower a person's chance of developing heart disease**	**True**	**73**.**1**	**86**.**5**	**0**.**02**
16. Diabetes is a risk factor for developing heart disease	True	84.5	90.4	0.41
17. High blood sugar puts a strain on the heart	True	78.9	86.5	0.20
18. If your blood sugar is high over several months, it can cause your cholesterol level to go up and increase your risk of heart disease	True	75.0	78.9	0.59
**19. A person who has diabetes can reduce their risk of developing heart disease if they keep their blood sugar levels under control**	**True**	**76**.**9**	**92**.**3**	**<0**.**01**
**20. People with diabetes rarely have high cholesterol**	**False**	**55**.**8**	**75**.**0**	**0**.**01**
**21. If a person has diabetes, keeping their cholesterol under control will help to lower their chance of developing heart disease**	**True**	**75**.**0**	**84**.**6**	**0**.**03**
**22. People with diabetes tend to have low HDL (good) cholesterol**	**True**	**7**.**7**	**25**.**0**	**0**.**03**
23. A person who has diabetes can reduce their risk of developing heart disease if they keep their blood pressure under control	True	78.9	84.6	0.26
24. A person who has diabetes can reduce their risk of developing heart disease if they keep their weight under control	True	80.8	84.6	0.32
25. Men with diabetes have a higher risk of heart disease than women with diabetes	False	32.7	36.5	0.56

*p*-values are from McNemar's test, bold values indicate statistical significance at the level of 0.05.

Heart disease risk factor knowledge improved in several domains, including family history of heart disease (question 2, *p* = 0.03), cholesterol (question 8, *p* = 0.02), and the difference between good and bad cholesterol (question 10, *p* = < 0.01 and question 11, *p* = 0.03). In addition, we observed improvements in knowledge regarding physical activity and the different types of physical activity that can prevent heart disease (questions 13–15, *p*-values all <0.05). When considering diabetes, knowledge increased regarding the association between blood glucose control and heart disease (question 19, *p* = < 0.01). Also, knowledge increased regarding the interaction between diabetes and cholesterol in the association with heart disease (questions 20–22, *p*-values all <0.05). On question 9, regarding the consumption of fatty foods and the effect on cholesterol, the percentage of correct responses decreased slightly from 86.5% to 75%, even though this material was covered in the presentation. However, this observation was not statistically significant (*p* = 0.11), indicating it could be due to chance. Finally, at baseline, only 33% correctly answered “false” to the question “Men with diabetes have a higher risk of heart disease than women with diabetes.” Since we did not focus on gender in our implementation program, the percentage of correct responses on this question did not increase on the post-survey (37%, *p* = 0.56).

## Discussion

The results of this study demonstrate that the “Amazing Race for Heart Health,” a shortened CVD risk factor educational program based on the HGHH curriculum, was effective at increasing knowledge regarding CVD risk factors. When comparing the pre- and post-surveys, the percentage of correct responses either increased or stayed the same. Likely due to the educational efforts by the SHS in the past, the participants had good baseline knowledge of several risk factors being associated with CVD, including age, smoking, high blood pressure, being overweight, and having diabetes. This observation provides an opportunity for future research about the effect of previous SHS outreach and educational efforts. However, even with this good baseline knowledge, the educational intervention that we developed was effective at improving CVD risk factor knowledge in several domains, including family history of heart disease, cholesterol, physical inactivity, and the difference between good and bad cholesterol. When considering diabetes, knowledge increased regarding the association between blood glucose control and heart disease. Also, knowledge increased regarding the interaction between diabetes and cholesterol in the association with heart disease. These observations are similar to previous reports on the knowledge improvements for the full HGHH program (18, 19, 26).

Although the results of this project provide valuable information about a culturally relevant educational intervention, there are some limitations. The beliefs expressed by the American Indians participating in this project are limited to seven communities in southwest Oklahoma, and therefore may not be transferrable or generalizable to other tribal communities. In addition, this was pilot study with a relatively small sample size consisting of mainly women. Despite these potential limitations, this project provides valuable insights on an interventional approach to address CVD prevention within American Indian communities. With this first step in program enhancement, we hope the methods presented can be used to adapt this interventional program for a larger audience, other tribal communities, or other groups with similar levels of documented cardiometabolic risk factors.

These results illustrate the value of health promotion and educational initiatives. Based on study results, we recommend that focus groups or knowledge surveys be conducted before HGHH or other interventions are implemented to identify areas where specific populations may lack awareness, and then the intervention be adapted for specific communities. In addition, these results indicate the value of an interactive component to reinforce the didactic approaches. In the current study population, the following areas may deserve more effort, the association between CVD and family history of heart disease (question 2), age as a CVD risk factor (question 3), cholesterol and the different types of cholesterol (questions 8–11), physical inactivity (questions 13–15), diabetes control (question 19), the interaction of diabetes and cholesterol (questions 20–22), and gender in CVD risk (question 25). For example, on question 22, “People with diabetes tend to have low HDL (good) cholesterol,” the percentage of correct responses increased significantly from 7.7% to 25%, but the percentage post-intervention remained relatively low. Therefore, we may need to include more information on the difference between “good” and “bad” cholesterol and how it may affect patients with diabetes. Finally, to address the potential misconception that there are gender differences regarding CVD risk among people with diabetes (question 25), we want to emphasize that both men and women with diabetes have an elevated risk of CVD. In conclusion, “Amazing Race for Heart Health,” a shortened CVD risk factor educational program based on the HGHH educational materials, was effective at increasing awareness regarding CVD risk factors and may be applicable in future research with American Indians.

## Data Availability

The data were collected, analyzed, and reported under agreements made with the sovereign tribal nations that have partnered in this research, which preclude commonly accepted modes of data sharing. Requests to access the dataset from qualified researchers trained in responsible conduct of research may be sent to the Strong Heart Study Coordinating Center at https://strongheartstudy.org/. Requests will be reviewed by tribal research partners before data may be released. This policy is consistent with the “NIH Policy for Data Management and Sharing: Responsible Management and Sharing of American Indian/Alaska Native Participant Data (https://grants.nih.gov/grants/guide/notice-files/NOT-OD-22-214.html#:∼:text=NIH%20recognizes%20that%20conducting%20biomedical,Tribes%5Biii%5D%20and%20communities).
